# Ring-Like Distribution of Constitutive Heterochromatin in Bovine Senescent Cells

**DOI:** 10.1371/journal.pone.0026844

**Published:** 2011-11-23

**Authors:** Andrey Pichugin, Nathalie Beaujean, Xavier Vignon, Yegor Vassetzky

**Affiliations:** 1 INRA UMR 1198 Groupe Biologie du Développement et Reproduction, Jouy en Josas, France; 2 ENVA UMR 1198 Groupe Biologie du Développement et Reproduction, Jouy en Josas, France; 3 Centre National de la Recherche Scientifique UMR 8126, Université Paris-Sud 11, Institut de Cancérologie Gustave-Roussy, Villejuif, France; Université Paris-Diderot, France

## Abstract

**Background:**

Cells that reach “Hayflick limit” of proliferation, known as senescent cells, possess a particular type of nuclear architecture. Human senescent cells are characterized by the presence of highly condensed senescent associated heterochromatin foci (SAHF) that can be detected both by immunostaining for histone H3 three-methylated at lysine 9 (H3K9me3) and by DAPI counterstaining.

**Methods:**

We have studied nuclear architecture in bovine senescent cells using a combination of immunofluorescence and 3D fluorescent in-situ hybridization (FISH).

**Results:**

Analysis of heterochromatin distribution in bovine senescent cells using fluorescent in situ hybridization for pericentric chromosomal regions, immunostaining of H3K9me3, centromeric proteins CENP A/B and DNA methylation showed a lower level of heterochromatin condensation as compared to young cells. No SAHF foci were observed. Instead, we observed fibrous ring-like or ribbon-like heterochromatin patterns that were undetectable with DAPI counterstaining. These heterochromatin fibers were associated with nucleoli.

**Conclusions:**

Constitutive heterochromatin in bovine senescent cells is organized in ring-like structures.

## Introduction

Somatic cells have a limited potential of proliferation. Normal human cells irreversibly enter a growth-arrested state called “replicative senescence” after a limited number of cell divisions *in vitro* [1], [2] that is caused by telomere shortening [3]. Senescent cells show a series of morphological and physiological alterations including a flat and enlarged morphology, an increase in acidic β-galactosidase activity [4] (senescence-associated β-galactosidase, SA-β-gal) as well as changes in the gene expression pattern [5]. Moreover, human senescent cells are characterized by chromatin condensation and formation of characteristic heterochromatin structures called senescence-associated heterochromatin foci (SAHFs). When stained with 4′-6-Diamidino-2-phenylindole (DAPI), young human cells exhibit a relatively even, diffuse distribution of DNA through the cell nucleus. However, in DAPI-stained senescent human cells, SAHFs appear as approximately 30–50 bright, punctate DNA foci [6]. Chromatin in these foci appears much more compact than the chromatin in normal interphase young cells and is more resistant to nuclease digestion [7]. Formation of highly condensed facultative heterochromatin includes the increase in HMGA and macroH2A levels as well as a higher level of histone H3 three-methylated at lysine 9 (H3K9me3) that lead to a more compact chromatin in SAHF [8], [9]. SAHFs are able to recruit proliferation-promoting genes, such as cyclin A2, into these compact chromatin foci thereby contributing to senescence-associated cell cycle arrest [6]. It is believed that the irreversible nature of human senescent cells is associated to alterations of chromatin structure [6], [10].

Indeed, it is now well established that the organization of nuclear compartments into repressed and active domains can play a major role in regulation of gene expression and is associated with cell type specialization. Cultured primary cells known as young cells usually display the so-called chromocenters composed of constitutive heterochromatin from satellite DNA of pericentric chromosomal regions that tend to cluster in interphase nucleus and provide a structural framework for the establishment of functional nuclear architecture [11], [12]. A dramatically different principle of nuclear organization in human senescent cells was initially described by appearance of SAHF [6] that do not represent such domains of constitutive heterochromatin. Centromeres, pericentric and telomeric chromosomal regions have been found at the periphery of SAHF [13], [14]. Human SAHFs contain several common markers of heterochromatin as CBX1, known as HP1beta, macroH2A and HMGA [7]. It has been shown that each SAHF in senescent cells results from condensation of an individual chromosome [13]. The earliest detectable event in the formation of a SAHF focus is the chromosome condensation, followed by methylation of lysine 9 of histone H3, binding of HP1 proteins and incorporation of macroH2A [7].

In the present work we have studied constitutive heterochromatin distribution in bovine cultured fibroblasts that reached proliferative senescence at late *in vitro* passages. We detected heterochromatin domains using a BAC probe specific for pericentric chromosomal regions of all bovine autosomes and using antibodies specific for histone H3 three-methylated at lysine 9 that is enriched in heterochromatic chromosomal domains. We also performed immunodetection of 5-methyl cytosine, CENP A/B as well as counterstaining with DAPI and YoPro1. We did not reveal any SAHF-like structures in senescent bovine fibroblasts. Instead, we observed fibrous distribution of constitutive heterochromatin that formed ribbon-like and ring-like structures associated with the nucleolar periphery.

## Results

Bovine primary fibroblasts were cultured *in vitro* for 25–34 passages. Replicative senescence of bovine fibroblasts was determined by terminal growth arrest (no population doubling within 2 weeks), morphological changes, i.e. an increase in cell size, an irregular shape, dark cytoplasmic granules and the proportion of senescent-associated beta-galactosidase-positive fibroblasts in non-confluent cultures. In order to detect senescence, we have stained cells with beta-galactosidase at each passage. Typical staining pattern is shown in Figure S1A. Next we have counted the proportion of beta-galactosidase-positive cells at each passage. The quantity of positively stained cells increased during passages (Figure S1B) in agreement with published data [16], [17], [18]. Starting from passage 24, cell cultures consisted of >95% cells positively stained with beta-galactosidase assay and had enlarged morphology (Figure S1). Cells that reached senescence could be cultivated up to passage 34 and be kept in culture for up to 8 weeks without apoptosis.

**Figure 1:**
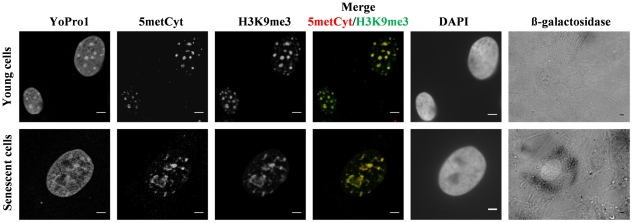
Distribution of heterochromatin domains in young and senescent bovine cells. Young cells from confluent culture of bovine fibroblasts (upper panel; n = 150) and senescent cells at late passages of the same culture (lower panel; n = 95) were immunostained to detect 5-methylcytosine (red) and H3K9me3 (green). Simultaneously, the nuclei were counterstained with YoProI and DAPI, and the cells were tested for β-galactosidase activity. Note the colocalization of H3K9me3 and DNA methylation in senescent and young cells. Images were obtained from confocal Z-stack projections. Scale bars: 5 µm.

Immunodetection of H3K9me3 and 5′-methylated cytosine showed clusters of heterochromatin in young (beta-galactosidase negative) cultured cells as normally observed in proliferating mammalian cells [19] (Figure 1 upper panel). In these young cells, heterochromatin clusters could also be observed with YoPro1 DNA counterstaining, while DAPI counterstaining did not allow to reveal heterochromatin clusters (Figure 1 upper panel; n = 150). In contrast, senescent cells (beta-galactosidase positive) displayed ring-like or ribbon-like fibers as shown by immunodetection of H3K9me3 and 5′-methylated cytosine or YoPro1 counterstaining (Figure 1 lower panel; n = 95). These structures were detected in a more than 80% of cells (n = 100) in cell cultures at late passages (>P24). DAPI counterstaining was distributed evenly both in these senescent cells and in young cells. We followed the nuclear organization in bovine cells to passage 34, well beyond the stage where more than 92% of the cells were beta-galactosidase positive, but we did not observe any SAHF-like structures.

**Figure 2:**
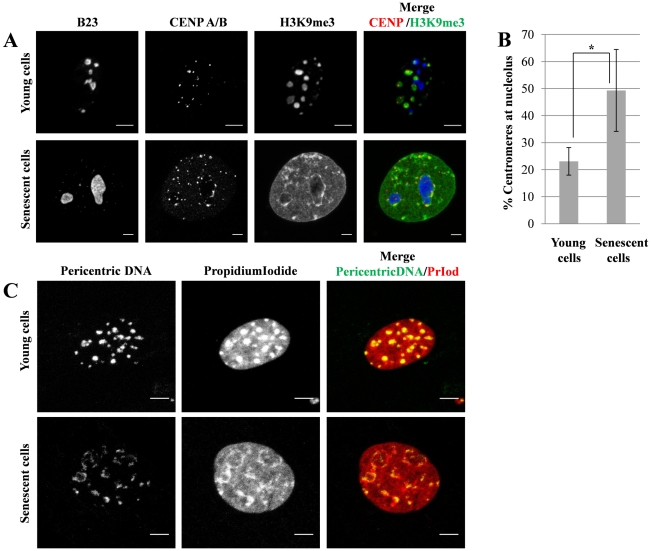
Centromeres and pericentric domains in nuclei of senescent bovine fibroblasts. (**A**) Confluent young primary bovine fibroblasts and cells at late passages were immunostained to detect CENP (red), H3K9me3 (green) and nucleoli (B23; blue). Compare the distribution of condensed heterochromatin (H3K9me3 distribution) in young cells and the distribution of dispersed ring- or ribbon-like heterochromatin that associates to nucleoli in senescent cells. Scale bars: 5 µm. (**B**) Quantitation of nucleoli-associated centromeres in senescent (n = 68) and young cells (n = 215). (**C**) Confluent primary young bovine fibroblasts (upper panel) and senescent cells (lower panel) labelled by fluorescent in situ hybridization with BAC231G08 (green) specific to pericentric regions of bovine autosomes and counterstained with Propidium iodide (red). Images are obtained from confocal Z-stack projections. Scale bars: 5 µm.

The clusters of heterochromatin observed in young cells correspond to chromocenters. Indeed, these heterochromatic domains detected by antibodies against H3K9me3 were always associated with chromosomal centromeres when co-detection for CENP A/B proteins was performed (Figure 2A upper panel; n = 215). Moreover, heterochromatin clusters were clearly observed by FISH with the BAC231G08 probe specific for pericentric chromosomal domains of autosomes (Figure 2C; n = 190). On the other hand, using these two approaches we did not detect true chromocenters in senescent cells. The ring- or ribbon-like heterochromatin distribution was observed also after FISH with BAC231G08 in these cells (Figure 2C; n = 54). The fibers detected with H3K9me3 were often associated with nucleoli (stained with B23; Figure 2A, lower panel; n = 68). Almost 50% of centromeres were also found to be associated with the nucleoli is senescent cells, as compared to approximately 20% in young cells (Figure 2B). At late passages, more than 80% of cells displayed ring-like distribution of H3K9me3 associated to nucleoli. This suggests that the fibrous heterochromatin observed in bovine senescent cells preferentially forms circular structures around nucleoli.

**Figure 3:**
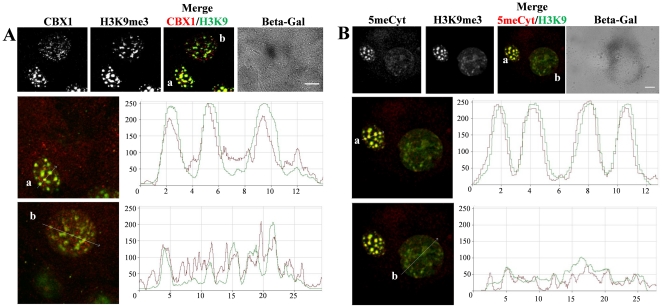
Heterochromatin markers in young and senescent bovine cells at passage 18. (**A**) Young and senescent cells exhibit different fluorescence intensity levels both H3K9me3 (green) and CBX1 (red) when compared in one optical field.Upper panel: Z-stack projection images after immunostaining of H3K9me3 and CBX1 and beta-gal assay. Scale bars: 5 µm. Middle panel (a): profiles of fluorescence intensities (right) of H3K9me3 (green) and CBX1 (red) mainly overlap when obtained from a single confocal section of the young cell nucleus. Lower panel (b): A senescent cell exhibits significantly lower levels of both CBX1 and H3K9me3 than a young cell. Horizontal and vertical axes correspond to micrometers and eight-bit fluorescence intensity levels, respectively. (**B**) Different levels of both H3K9me3 (green) and 5-methylcytosine (red) in young (a) and senescent (b) cells. Upper panel: Z-stack projection images of young (a) and senescent (b) cells were immunostained for H3K9me3 and 5-methylcytosine and captured in one optical field. Scale bars: 5 µm. Middle (a) and lower (b) panels: profiles of fluorescence intensities (right) of H3K9me3 (green) and 5-methylcytosine (red) obtained from a single confocal section of the nucleus in a young cell (left; a) and a senescent cell (left; b). Note significantly lower levels of both marks in the senescent cell as compared to the young cell.

In order to directly compare the senescent and proliferating cells, we used cells at passage 18 where senescent (beta-Gal-positive) cells can be found along with proliferating (beta-galactosidase negative) ones. Senescent cells usually had two-fold decrease in the levels of fluorescence intensity for each 5′-methylcytosine, CBX1 (HP1beta) and H3K9me3 staining as compared to proliferating cells (Figure 3, lower panel). This difference was observed in senescent cells both with the large (Figure 3) and the normal nucleus size (data not shown). The levels of CBX1, another heterochromatin marker normally recruited by H3K9me3 [20], were also significantly lower in senescent cells as compared to proliferating ones (Figure 3A and Figure S2; n = 75). These data demonstrated that senescence-associated structural reorganization of heterochromatin in bovine cells was related to decondensation and was simultaneous with a decrease in CBX1 levels.

**Figure 4:**
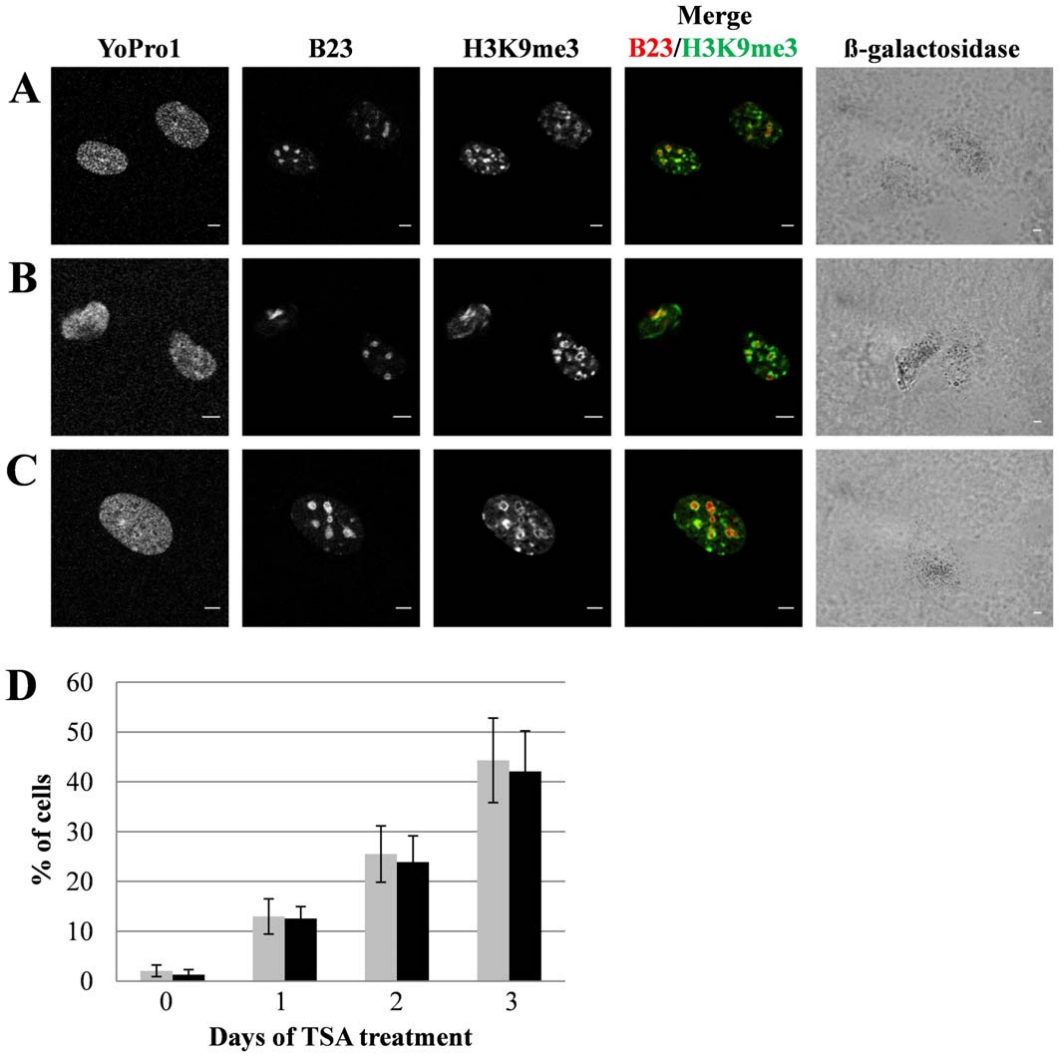
Induction of senescence and nuclear distribution of heterochromatin in primary bovine fibroblasts after treatment with trichostatin A (TSA). Young bovine fibroblasts at passages 2–3 were treated with 1 mM TSA for 1, 2, or 3 days (**A–C**) to induce senescence and then were stained for B23, H3K9me3 and beta-galactosidase. Note that the H3K9me3 (green) pattern has a high intensity and is distributed around nucleoli (B23; red). Scale bars: 5 µm. (**D**) The percentage of beta-galactosidase-positive cells (gray bars) and cells with the ring-like chromatin distribution (black bars) was counted at 0, 1, 2 and 3 day of TSA treatment (n = 200 for beta-galactosidase staining and n = 80 for immunofluorescence for each experimental point; the results of two independent experiments are shown).

Next, we have treated the primary bovine fibroblasts with deacetylase inhibitor trichostatin A (TSA) to induce senescence and obtained the ring-like pattern of heterochromatin distribution in the majority of treated cells. The number of beta-galactosidase-positive cells in this experiment correlated with the number of cells with the ribbon-like chromatin structures, but we did not observe any SAHF-like structures in these conditions either (Figure 4, n = 160).

## Discussion

### Senescent bovine cells form ribbon-like and ring-like heterochromatin structures

Both young and aging bovine fibroblasts might be used for nuclear transfer into oocytes for research and cloning purposes. Surprisingly, aging bovine fibroblasts show higher nuclear transfer and developmental efficiency as compared to young cells [21] therefore it is essential to study aging and senescence in bovine cells. Human senescent cells form highly specific heterochromatin structures, SAHFs. Here we have demonstrated that bovine senescent cells also display a profoundly reorganized heterochromatin structure as compared to young (proliferating) bovine cells, as detected using immunostaining for H3K9me3, 5-methyl cytosine, CENP, CBX1 and pericentric satellite sequences. While primary bovine fibroblasts possessed globular blocks of constitutive heterochromatin, known as chromocenters and typical of mammalian cells, the senescent cells had ribbon-like and ring-like heterochromatin structures also formed from constitutive heterochromatin, which are dramatically different from the human and murine SAHFs. These ribbon-like heterochromatin structures often associate with the periphery of nucleoli. The comparative study of distribution of epigenetic marks and pericentric satellite sequences in young and senescent bovine cells revealed a significant decondensation of constitutive heterochromatin in senescent cells. To our knowledge, these are the first observations of a species-specific senescence-associated nuclear architecture.

Formation of senescence associated heterochromatin foci (SAHFs) is characteristic for human and, to a lesser extent, murine cells. It is an essential event for proliferative arrest of cells which undergo induced or native senescence [22]. We could not observe SAHFs in bovine cells using neither DAPI nor other counterstaining. A homogeneous DAPI staining was observed in nuclei of both young and senescent cells (Figure 1 lower panel). At the same time, YoPro1 (Figure 1) or Propidium Iodide (Figure 2C) counterstaining revealed blocks of constitutive heterochromatin in young cells and unexpected ribbon- and ring-like structures in senescent cells. This may be explained by high specificity of DAPI for AT-rich DNA sequences [23]; bovine pericentric heterochromatin has a GC-rich content, therefore heterochromatin patterns could not be detected by DAPI.

We stained heterochromatin in young and senescent cells using antibodies against tri-methylated lysine 9 of histone H3 (H3K9me3). It specifically labels chromocenters in interphase nuclei of proliferating mammalian cells and reveals SAHF in human senescent cells [9], [24]. The H3K9me3 marks mainly co-localized with YoPro1 counterstaining in both cell types (Figure 1). Young cells displayed globular blocks of constitutive heterochromatin that associated with centromeres (Figure 2A) forming structures specific for mammalian cells and known as chromocenters. Both H3K9me3 and YoPro1 staining were co-localized in senescent cells at ring-/ribbon-like structures (Figure 1), thus confirming that SAHFs are absent in bovine senescent cells.

### The origin of ring-/ribbon-like heterochromatin in bovine senescent cells

The SAHFs in human cells derive from condensation of individual chromosomes [13], [14]. They associate with blocks of constitutive heterochromatin formed from satellite sequences [6], [14], [25]. In order to follow the distribution of constitutive heterochromatin domains in interphase nuclei, we used 3D FISH with a probe specific to pericentric chromosomal regions. The signals from satellite sequences and from Propidium Iodide were co-localized in both young and senescent cells (Figure 2C). The constitutive heterochromatin detected with this probe formed chromocenters in young cells and ring-/ribbon-like heterochromatin in senescent cells (Figure 2), suggesting that ring-/ribbon-like heterochromatin distribution in senescent cells derives from reorganized constitutive heterochromatin of chromocenters.

Heterochromatin protein 1 beta (CBX1) drives chromocenters formation in young cultured mammalian cells as well as participates in SAHF [20], [26]. CBX1 staining was strongly co-localized with H3K9me3 in highly condensed constitutive heterochromatin of bovine young cultured cells (Figure 3A). In contrast, the senescent cells had a weaker fluorescence intensity of CBX1 (Figure 3A and Figure S2) that mainly co-localized with H3K9me3. The fluorescence intensity of 5′-methyl cytosine and H3K9me3 was also significantly lower in senescent cells as compared to young cells (Figure 3B). These results are in agreement with the previous observations [27], [28], [29] but the decrease in fluorescence intensity of H3K9me3 and CBX1 in bovine senescent cells appears different to those in human senescent cells [6], [9], [26]. Thus, Zhang and colleagues observed that abundant CBX1 proteins are not required for the formation of SAHF in human cells [14]. Therefore it should be tested whether heterochromatin decondensation in bovine senescent cells is associated with reduced CBX1 levels. Ring-like heterochromatin and centromeric regions associated with the periphery of nucleoli (Figure 2), suggesting a significant reorganization of chromocenters. The TSA-induced senescent cells also exhibited ring-like heterochromatin structure around nucleoli (Figure 4). Hence, both proliferative and induced senescence in bovine cells leads to heterochromatin association with nucleoli. Indeed, nucleoli are associated with heterochromatin [30] and they may play a role of heterochromatin organizers similar to SAHF during senescence in bovine cells. Perinucleolar localization has also been shown for PML bodies which often form ring-like structures around nucleoli in human senescent cells [31], [32]. In agreement with the previous data [22], [33] we have found a significant increase in the size and number of PML bodies in bovine senescent cells (data not shown), although we were not able to observe their association with nucleoli. Interestingly, translocation of phosphorylated histone chaperone protein (HIRA) to PML bodies is a pre-requisite for HIRA to promote formation of punctate SAHFs in human cells [14], [22]. Further studies will be required to fully define the mechanism of heterochromatin reorganization in bovine senescent cells.

## Methods

### Cell cultures

Bovine primary fibroblasts cell line OV5538 [15] was cultured at 39°C in humidified 5% CO2 atmosphere in Dulbecco modified Eagle's medium (DMEM) supplemented with 10% fetal calf serum and penicillin-streptomycin (100 Units/mL penicillin, 100 µg/mL streptomycin; Gibco). Each time when the culture reached 95% to 100% confluence, cells were split at a 1∶8 ratio. Passages of cell cultures were performed by treatment with a trypsin/PBS solution during 5–10 min at 37°C in humidified 5% CO2 atmosphere, supplementation of DMEM and centrifugation during 5 min at 1000 rpm. Pellet was then suspended in DMEM and transferred in 5 cm diameter Petri dishes with 5 ml DMEM supplemented solution. Cultures were grown until the onset of senescence (24–34 passages). For cytological analysis cells were subcultured on glass slides (Superfrost) using Flexiperm silicon wall.

### Cytochemical acidic β-Galactosidase Assay

Confluent and senescent cells were washed in PBS, fixed in 2% formaldehyde/0.2% glutaraldehyde/PBS for 3–5 min at room temperature, washed in PBS, and incubated at 37°C in freshly prepared X-gal staining solution (1 mg/ml 5-bromo-4-chloro-3- indoyl b-D-galactopyranoside), 40 mM citric acid/Na phosphate buffer (pH 6.0), 5 mM potassium ferricyanide, 5 mM potassium ferrocyanide, 150 mM NaCl, and 2 mM MgCl2 for 16 h. The stained cells were detected and images captured on a Zeiss Axiovert microscope at 20× magnification.

### Immunostaining

After fixation in 2% paraformadehyde in PBS for 30 min, the cells were washed for 10 min in PBS and permeabilized by 0.5% Triton X- 100/PBS solution during 30 min at room temperature (RT). Cells were then incubated at RT during 1 hr in 2% BSA/PBS (BSA: bovine serum albumin) to saturate non-specific binding sites. Incubation with the primary antibodies diluted in 2% BSA/PBS was performed during 1 hr at 37°C or overnight at 4°C in 30 µL antibodies drops under Parafilm in a humid box. Then the cells were washed twice (15 min each, RT) in PBS and incubated with the secondary antibodies, diluted in 2% BSA/PBS, during 1 hr at RT. After immunostaining the cells were washed for 30 min in PBS at RT, counterstained with YoPro1 and mounted on glass slides with DAPI-containing antifading medium (Vectashield; Vector laboratories, H-1200). Glass coverslips were sealed with nail polish. H3K9me3 was detected with primary rabbit monoclonal anti-H3K9me3 antibody (Millipore, 07-523) diluted 1∶200 in 2% BSA/PBS and donkey Cy5-conjugated anti-rabbit secondary antibody (Jackson ImmunoResearch, 711-175-152) diluted 1∶100 in 2%BSA/PBS. B23 protein was detected with the primary mouse monoclonal anti-nucleophosmin antibody clone FC-619991 (Zymed, Ref 32-5200), diluted 1∶200 in 2% BSA/PBS and donkey rhodamine isothiocyanate (TRITC) -conjugated anti-mouse secondary antibody (Jackson Immunoresearch, 715-025-150) diluted 1∶100 in 2%BSA/PBS. The centromeres were labeled with primary human CREST antibody specific to CENP-A and CENP-B (Immunovision, HCT-0100) diluted 1∶200 in 2%BSA/PBS and FITC-conjugated anti human secondary antibody (Jackson Immunoresearch, 709-095-149) diluted 1∶100 in 2% BSA/PBS. CBX1 protein was detected with the primary mouse anti- CBX1 MAB clone 1 MOD 1A9 (Euromedex), diluted 1∶200 in 2% BSA/PBS and TRITC-conjugated anti-mouse secondary antibody (Jackson Immunoresearch, 715-025-150) diluted 1∶100 in 2% BSA/PBS.

### 5-Methylcytosine Immunodetection in Cells

Cells were washed in PBS and fixed for 20 min at room temperature in 2% paraformaldehyde. Cells were washed extensively in PBS before further processing, then permeabilized with 0.5% Triton X-100 for 30 min at room temperature and treated with 4 N HCl for 1 h at 37°C. After several washes with 0.05% Tween-20, cells were blocked for 1 h in PBS containing 2% BSA (PBS-BSA 2%). Methylated DNA was visualized with a mouse monoclonal antibody against 5-methylcytosine (5-mC) (Eurogentec, BI-MECY-0100) simultaneously with anti-H3K9me3 antibodies. Incubation with these antibodies was performed at 37°C for 1 h (1∶200 dilution in PBS-BSA 2%), followed by washes with PBS (30 min) and 1 h incubation at room temperature with a TRITC-conjugated anti-mouse secondary antibody (1∶200 dilution; Jackson Immunoresearch, 715-025-150). After several washes in PBS, nuclear DNA was counterstained with YoPro1 (Invitrogen, Y3603) and DAPI and cells were mounted in Vectashield.

### Three-dimensional fluorescence in situ Hybridization (3D-FISH) with a pericentric probe

Cells were fixed in 4% paraformaldehyde in 1×PBS, permeabilized with 0.5% Triton X-100, incubated in 20% glycerol, repeatedly frozen-thawed using liquid nitrogen, incubated in 0.1 N HCl for 5 min, and stored in 50% formamide, 2×SSC at 4°C until hybridization. A probe BAC231G08 contained in sequences from pericentric regions of all bovine autosomes was obtained from bovine BAC library of INRA, Jouy en Josas. Following labeling with biotin-14-ATP by nick-translation the probe was shortened to 100–500 bp by DNase digestion. The probe was dissolved in hybridization mixture (50% formamide, 10% dextran sulfate, 1×SSC) to the concentration 20 ng/µl, loaded on a coverslip with cells, covered with a smaller coverslip, and sealed with rubber cement. Cell and probe DNA were denatured simultaneously on a hot-block at 75°C for 4 min. Hybridization was performed for 1 day at 37°C in a humid box. Post-hybridization washes were done in 2×SSC at 37°C and 0.1×SSC at 60°C. Nuclear DNA was counterstained with Propidium Iodide and cells were mounted in Vectashield.

### Confocal and fluorescent microscopy

Cells were observed under a Zeiss LSM 510 confocal microscope (MIMA2 Platform, INRA), using a Plan-Apochromat 63×1.4 oil objective. Imaging was performed with sequential multitrack scanning using the 488, 543 and 633 nm wavelengths lasers separately. The Z-stacks were acquired using a frame size of 512×512 with a pixel depth of 8 bits and 0.37 µm z-steps. Images of DAPI staining and SA-β-gal assay were obtained at the same microscope with an AxioCam HRm (Zeiss) camera.

## Supporting Information

Figure S1 **Senescent cells in** ***in vitro*** **cultures of bovine fibroblasts.** (**A**) Cultured cells were tested for acidic β-galactosidase activity and stained with hematoxyline. Senescent cells at passages 4 (P4), 16 (P16) and 26 (P26) stained in blue are indicated by arrows. (**B**) The number of senescent cells at each passage was estimated by counting the percentage of β-galactosidase positive cells. 300 cells were counted at each passage. The data represent the average of three independent experiments.
(TIF)

Figure S2 **Decondensed constitutive heterochromatin in senescent bovine cells obtained from the cell culture beyond passage 30.** Note a low level of CBX1 mark and a homogeneous distribution of both H3K9me3 (blue) and CBX1 (red) in senescent cell nuclei. Staining: YoPro1 (green), CBX1 (red), H3K9me3 (blue), DAPI counterstaining and beta-galactosidase assay. Scale bars: 5 µm.
(TIF)
