# Supplementary figures and images for: Ring-Like Distribution of Constitutive Heterochromatin in Bovine Senescent Cells
Source: PLoS One. 2011 Nov 23;6(11):e26844. doi: 10.1371/journal.pone.0026844 (PMC3223162; doi:10.1371/journal.pone.0026844)

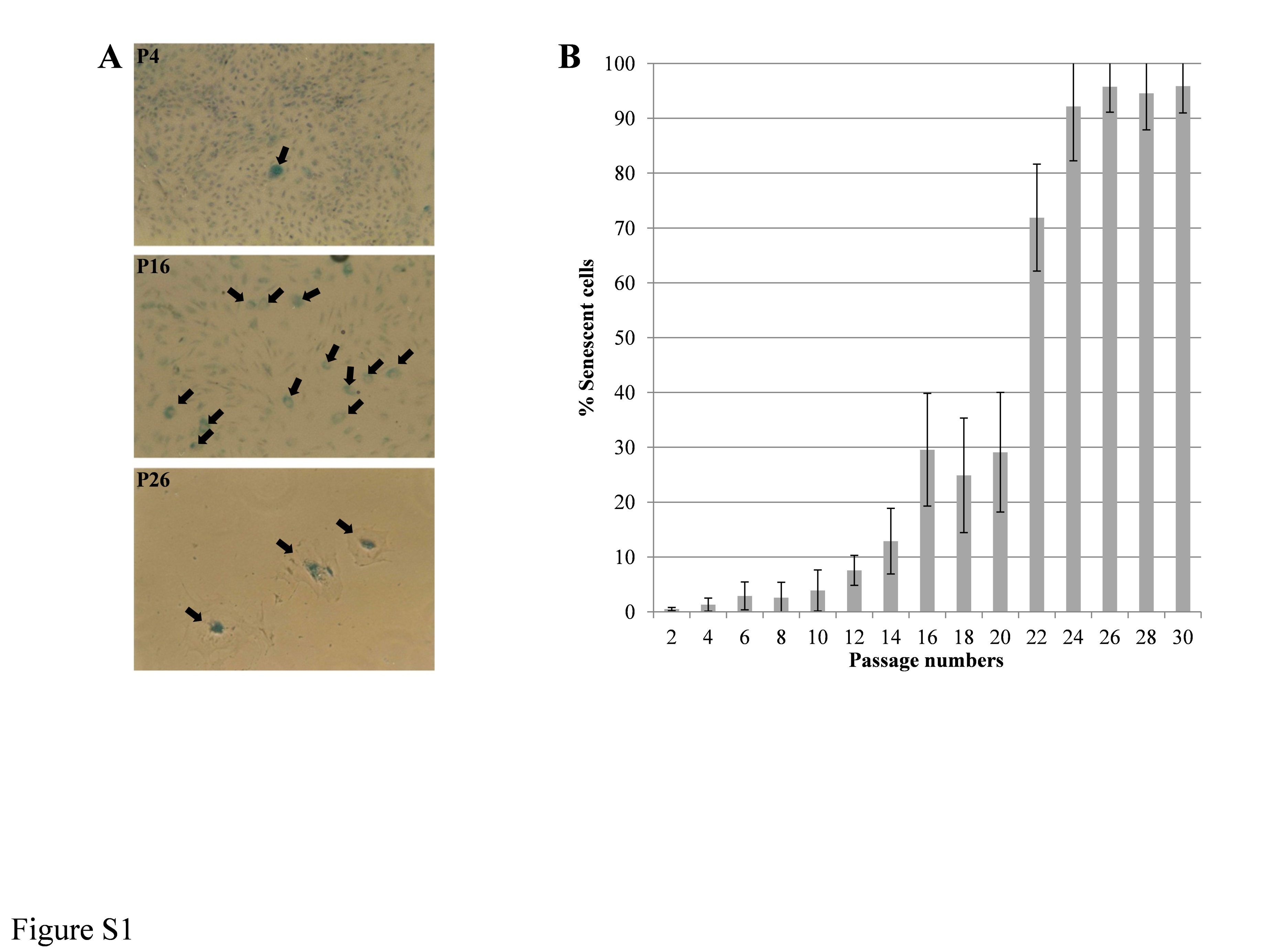

Supplement: Figure S1 — Senescent cells in in vitro cultures of bovine fibroblasts. (A) Cultured cells were tested for acidic β-galactosidase activity and stained with hematoxyline. Senescent cells at passages 4 (P4), 16 (P16) and 26 (P26) stained in blue are indicated by arrows. (B) The number of senescent cells at each passage was estimated by counting the percentage of β-galactosidase positive cells. 300 cells were counted at each passage. The data represent the average of three independent experiments. (TIF) [file pone.0026844.s001.tif]

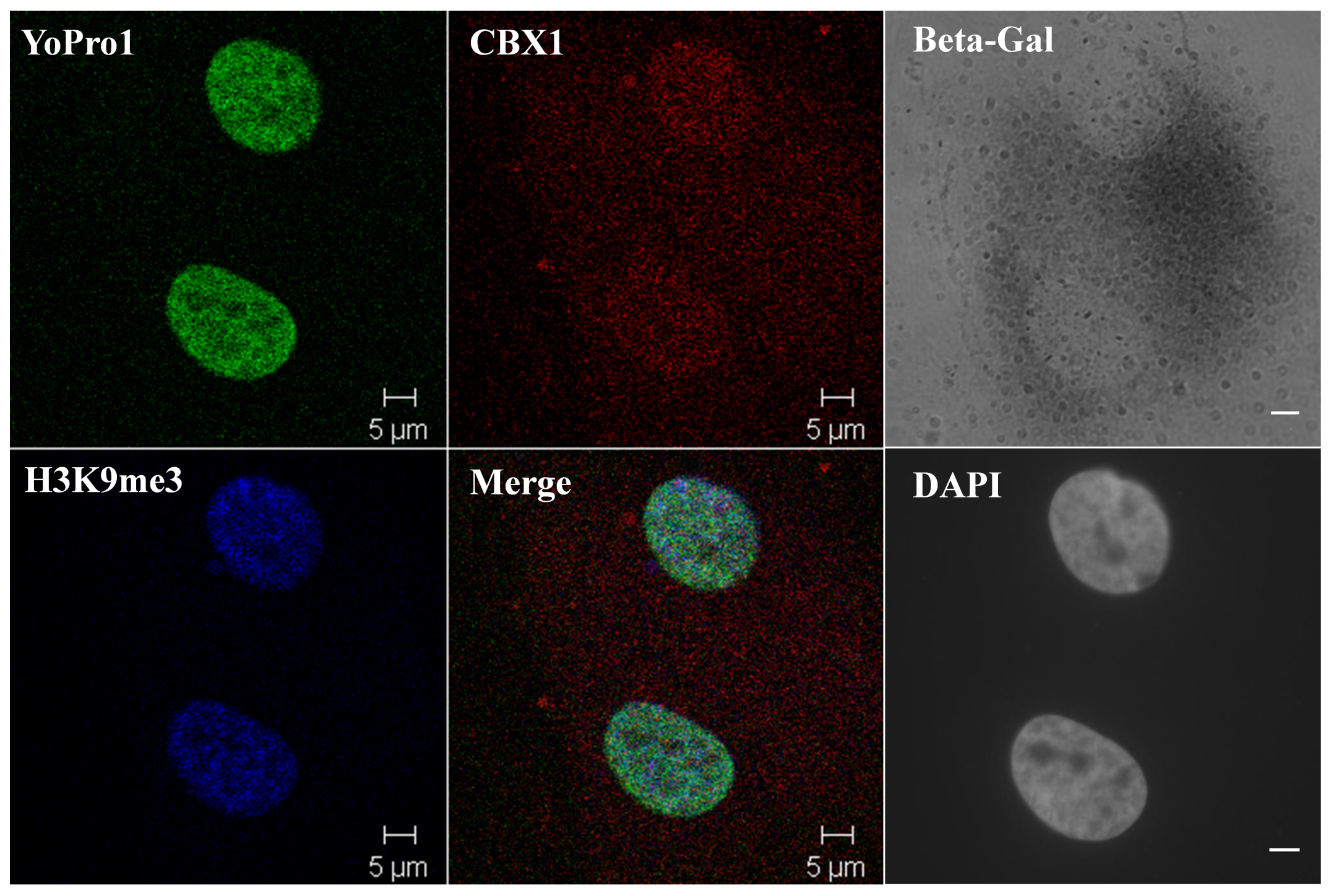

Supplement: Figure S2 — Decondensed constitutive heterochromatin in senescent bovine cells obtained from the cell culture beyond passage 30. Note a low level of CBX1 mark and a homogeneous distribution of both H3K9me3 (blue) and CBX1 (red) in senescent cell nuclei. Staining: YoPro1 (green), CBX1 (red), H3K9me3 (blue), DAPI counterstaining and beta-galactosidase assay. Scale bars: 5 µm. (TIF) [file pone.0026844.s002.tif]
